# Things are not always what they seem: Histopathology of Covid‐19 lymphadenopathy

**DOI:** 10.1002/jha2.156

**Published:** 2021-01-26

**Authors:** Catherine M. Tucker, Guldeep K. Uppal

**Affiliations:** ^1^ Department of Pathology, Anatomy, and Cell Biology Thomas Jefferson University Hospital Philadelphia Pennsylvania

**FIGURE 1:**
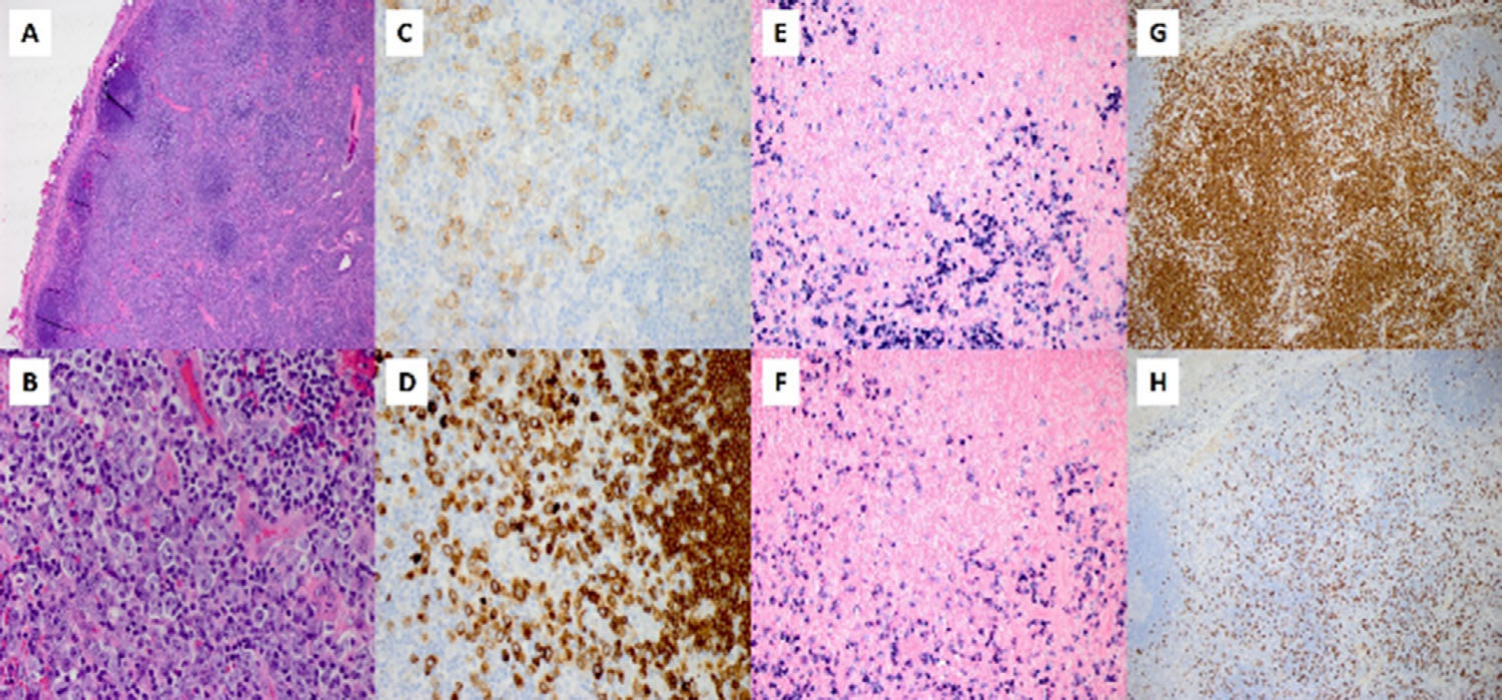
A. Nodal architecture at low power with atypical paracortical hyperplasia; hematoxylin and eosin stain, objective 4x. B. Paracortex with abundant immunoblasts; hematoxylin and eosin stain, objective 50x. C and D. CD30 and CD79a, respectively, highlighting immunoblasts; objective 40x. E and F. In situ hybridization staining for kappa and lambda; objective 20x. G and H. CD4 and CD8, respectively, with a ratio of greater than 10:1; objective 10x.

A 58‐year‐old female presented with respiratory failure due to Covid‐19. She underwent a tracheostomy procedure and concurrent excisional biopsy of a left neck level 6 lymph node. The lymph node was 0.9 cm in dimension with preserved nodal architecture and atypical paracortical hyperplasia (Figure 1, panel A; H&E stain, objective 4x). The paracortex showed abundant immunoblasts, small lymphocytes, and a few plasma cells (Figure 1, panel B, H&E stain, objective 50x). Scattered residual lymphoid follicles were noted in the cortex. Immunoblasts expressed CD30 (Figure 1, panel C; membranous and Golgi staining, objective 40x), CD79a (Figure 1, panel D; objective 40x), PAX‐5, CD138, and downregulation of CD20 (variable dim staining in a subset). They showed polytypic light chain expression by in situ hybridization staining for kappa and lambda (Figure 1, panels E and F; objective 20x). The paracortical T cells were mostly helper T cells with a CD4 to CD8 ratio of greater than 10:1 (Figure 1, panels G and H; objective 10x).

Detailed pathologic findings of excisional lymph node biopsies in patients with Covid‐19 have not yet been reported in the literature. Unique features of this case include the florid polyclonal immunoblastic proliferation despite no lymph node enlargement, and the lack of follicular hyperplasia. The patient is currently in remission.

